# Application of enhanced recovery after surgery in laparoscopic biliary reoperation for extrahepatic bile duct stones

**DOI:** 10.3389/fsurg.2026.1738211

**Published:** 2026-02-16

**Authors:** Changjie Lin, Zhihong Jiang, Linjun Zhuang, Shaohua Wei, Yecheng Li, Xiaoming Ma

**Affiliations:** 1 Department of General Surgery, The Second Affiliated Hospital of Soochow University, Suzhou, Jiangsu, China; 2 Department of General Surgery, Suzhou Industrial Park XingTang Hospital, Suzhou, Jiangsu, China

**Keywords:** biliary reoperation, eras, extrahepatic bile duct stones, laparoscopic surgery, postoperative recovery

## Abstract

**Objective:**

To evaluate the safety and efficacy of enhanced recovery after surgery (ERAS) protocols in laparoscopic biliary reoperations for extrahepatic bile duct stones.

**Methods:**

A total of 60 patients with prior biliary surgery were randomly assigned to either an ERAS group or a control group (*n* = 30 each). Both groups underwent laparoscopic bile duct exploration. Perioperative outcomes, inflammatory markers (CRP, IL-6), and nutritional indicators (prealbumin) were compared. Functional recovery, quality of life (SF-36), and complications were also assessed.

**Results:**

The ERAS group showed significantly shorter time to first flatus, faster ambulation and oral intake, lower pain scores, shorter hospital stays, and reduced hospitalization costs (*P* < 0.05). Patient satisfaction at discharge and 3 months postoperatively was significantly higher. SF-36 scores at 4 weeks showed better physical function, vitality, and general health in the ERAS group. CRP and IL-6 levels were lower, and prealbumin levels were higher postoperatively in the ERAS group (*P* < 0.05). The incidence of complications such as severe nausea and vomiting was lower in the ERAS group.

**Conclusions:**

ERAS is a safe and effective strategy in laparoscopic reoperation for extrahepatic bile duct stones. It significantly improves early recovery, reduces inflammation and costs, enhances patient satisfaction, and supports wider adoption of ERAS in hepatobiliary surgery.

## Introduction

Enhanced recovery after surgery (ERAS), proposed by Danish surgeon Kehlet (1), is a perioperative improvement strategy aimed to minimize the stress response and organ dysfunction to accelerate postoperative recovery and reduce surgery-related complications.

In general surgery, the ERAS concept was initially applied in colorectal resection, which was recognized by the academic community for its ability to significantly shorten length of hospital stay (2). In recent years, the concept has been widely adopted in other departments, including orthopedics, thoracic surgery, obstetrics and gynecology, and urology. Laparoscopic technique has been widely used in hepatolithiasis surgery due to its less trauma and rapid postoperative recovery. With the advancements in surgical techniques, the indications for laparoscopic surgery have expanded, and a history of biliary surgery is no longer considered a contraindication. China is a high-incidence area for bile duct stones. Extrahepatic bile duct stones are characterized by complex conditions, high rates of residual/recurrent stones and multiple complications. Recurrent cholangitis caused by extrahepatic bile duct stones severely affects the quality of life of patients and exacerbates the economic burden on the healthcare system in China (3). Currently, surgical intervention remains the mainstream treatment for extrahepatic bile duct stones, and it is common for patients to undergo reoperation due to stone recurrence.

Although the ERAS concept has gradually been applied in hepatobiliary and pancreatic surgery, it has not yet been widely adopted. Particularly, there is still no consensus among experts, both domestically and internationally, on ERAS perioperative management strategies for reoperations involving extrahepatic bile duct stones. Many surgeons still believe that the impacts of first surgery often lead to severe intra-abdominal adhesions during reoperations, potentially resulting in inadvertent injury to the bile ducts or even the intestines. Consequently, surgeons often tend to opt for open surgery for reoperations to reduce the occurrence of surgical complications. However, as laparoscopic techniques have been introduced in China and have advanced over several decades, reoperations are no longer considered a contraindication for laparoscopic surgery. Data suggest that there is no significant difference in complications, such as bile duct injury, intestinal injury, and bile leakage, between open surgery and laparoscopic surgery, but laparoscopic surgery offers clear advantages in early postoperative ambulation, eating, and shorter length of hospital stays (4–6).

However, there is still insufficient evidence to support the application of the ERAS concept in laparoscopic biliary reoperation. In 2018, the Chinese Expert Consensus on ERAS also pointed out that laparoscopic surgery should be the first choice for suitable patients under strict indication control and with adequate consideration of equipment, technique, and patient conditions (7). Therefore, we attempted to apply the ERAS concept to the perioperative management of laparoscopic biliary reoperations for extrahepatic bile duct stones. Additionally, we conducted a randomized controlled trial comparing this strategy with the traditional perioperative management strategy of the same period to observe its clinical application effects.

## Study subjects and methods

### Study subjects

The study included 60 patients with extrahepatic bile duct stones who underwent surgical treatment at our hospital between January 2021 and December 2024. The clinical data for all patients were complete, with a history of biliary surgery. The inclusion criteria for enrolled patients were as follows: (1) presence of extrahepatic bile duct stones confirmed by magnetic resonance cholangiopancreatography; (2) history of prior biliary surgery, including cholecystectomy or bile duct exploration; (3) inability to remove the stones once via ERCP, as confirmed by a gastroenterologist; and (4) dilation of extrahepatic bile duct, with a diameter > 8 mm. The exclusion criteria included: (1) severe cardiopulmonary dysfunction; (2) history of biliary-enteric anastomosis; and (3) conversion to open surgery during the procedure.

Patients were randomly divided into the ERAS group and the control group, with 30 patients in each group, using block randomization. The random numbers were generated by a computer, with a 1:1 allocation ratio. The sealed envelopes containing random numbers, prepared by the study designers, were distributed to clinical physicians, ensuring that the entire experimental process was conducted without any direct contact with the patients. The clinical doctors responsible for patient enrollment, intervention, and result analysis were not involved in the randomization process. The patients in the ERAS group were treated with optimized measures during the perioperative period, while the control group received traditional management.

### Blinding

Blinding of patients and treating clinicians was not feasible due to the clear differences between the perioperative management strategies. To minimize potential subjective bias, outcome assessment and statistical analysis were conducted independently by investigators who were not involved in patient care.

### Ethical approval

This study was conducted in accordance with the Declaration of Helsinki. The research protocol was reviewed and approved by the Ethics Committee of the Second Affiliated Hospital of Soochow University (Approval No. JD-LK202047-101, Project No. LK2020047).

This study was approved by the Ethics Committee of the Second Affiliated Hospital of Soochow University, and informed consent was obtained from all enrolled patients before participation.

### Study methods

The perioperative management plans for patients in the ERAS and control groups are outlined in Table 1.

**Table 1:** Perioperative management plans in the ERAS and control groups.

Intervention	ERAS group	Control group
Preoperative education	Routine admission education and explanation of the entire ERAS concept and process, psychological care to alleviate patient concerns, and a brief introduction to the treatment plan	Routine admission education
Preoperative fasting	Solid food prohibition 6 h preoperatively, with the administration of 10% glucose solution (300 mL) 2 h before surgery	Preoperative fasting for 12 h and water restriction for 6 h before surgery
Preoperative bowel preparation	Lack of routine bowel preparation	Routine administration of a soap suds enema the night before surgery to prevent bowel injury and facilitate repair if necessary
Nasogastric tube placement	Not routinely placed. Temporary gastrointestinal decompression when severe gastric distention is observed during surgery for better surgical field exposure, with the tube removed immediately after surgery	Routine preoperative placement, with removal upon the patient's first flatus
Urinary catheter placement	Placement after general anesthesia and removal within 24 h postoperatively	Routine placement, with intermittent clamping on the first postoperative day and bladder function training for 3 days to remove the catheter if the patient reports the desire to urinate
Intraoperative anesthesia management	General anesthesia with endotracheal intubation. Local infiltration anesthesia with 5 mL of 2% lidocaine at each incision site before skin incision, followed by intravenous administration of 5 µg fentanyl before skin closure; controlled intraoperative infusion at 4–6 mL/(kg·h); enhanced warming measures during surgery to maintain body temperature >36°C, with real-time monitoring of patient temperature and room temperature	Routine general anesthesia with endotracheal intubation; routine intraoperative infusion at 8–10 mL/(kg·h); no specific warming measures for the patient during the surgery, with operating room temperature maintained at 22–25 °C.
Surgical methods	Laparoscopic common bile duct exploration + T-tube drainage; primary suture of the common bile duct for treating patients with some common bile duct diameter > 10 mm, few stones, no residual stones confirmed by choledochoscopy, and no edema or stricture of the duodenal papilla; requiring conversion to open surgery	Laparoscopic common bile duct exploration + T-tube drainage
Drainage tube placement	Routine placement of one tube	Routine placement of one tube
Postoperative analgesia	Planned multimodal analgesia using scheduled non-opioid agents (intravenous NSAIDs and/or oral acetaminophen), with patient-controlled intravenous opioid analgesia available as needed	Standard stepwise analgesia with non-opioid medications (intravenous NSAIDs and/or oral acetaminophen/ibuprofen) as first-line therapy, with opioid analgesics used only as rescue when pain remained insufficiently controlled
Postoperative diet	Small amounts of water allowed 6 h postoperatively, a liquid diet on the first postoperative day, progressing to a semi-liquid diet and eventually returning to a normal diet.	Small amounts of water only after the patient's first flatus, postoperatively, gradually returning to a normal diet
Drainage tube removal	Removal of the drainage tube on the second postoperative day if there is no active bleeding, bile leakage, or significant exudation	Removal of the drainage tube when drainage fluid was < 50 mL, the nature of the fluid was normal, and no intra-abdominal fluid accumulation was confirmed by ascites ultrasound
Postoperative activity	Encouragement for early ambulation on the first postoperative day, with an initial activity duration of 30 min, gradually increasing daily	No mandatory early mobilization; activity duration determined by the patient's condition

All patients underwent related preoperative examinations, including blood routine, coagulation series, biochemical complete set, electrocardiogram, chest x-ray, and magnetic resonance cholangiopancreatography to confirm the diagnosis. Additionally, the overall condition of patients was evaluated, and necessary adjustments were made to optimize their physiological status to meet surgical requirements. Most patients presented with various symptoms, such as upper abdominal discomfort, nausea, and vomiting, with some exhibiting varying degrees of jaundice. The preoperative examinations by the clinician confirmed that patients meeting the inclusion criteria were selected for the study. The American Society of Anesthesiologists score of patients assessed by anesthesiologists ranged from I to II, indicating that they were eligible for surgical treatment.

**Figure 1:**
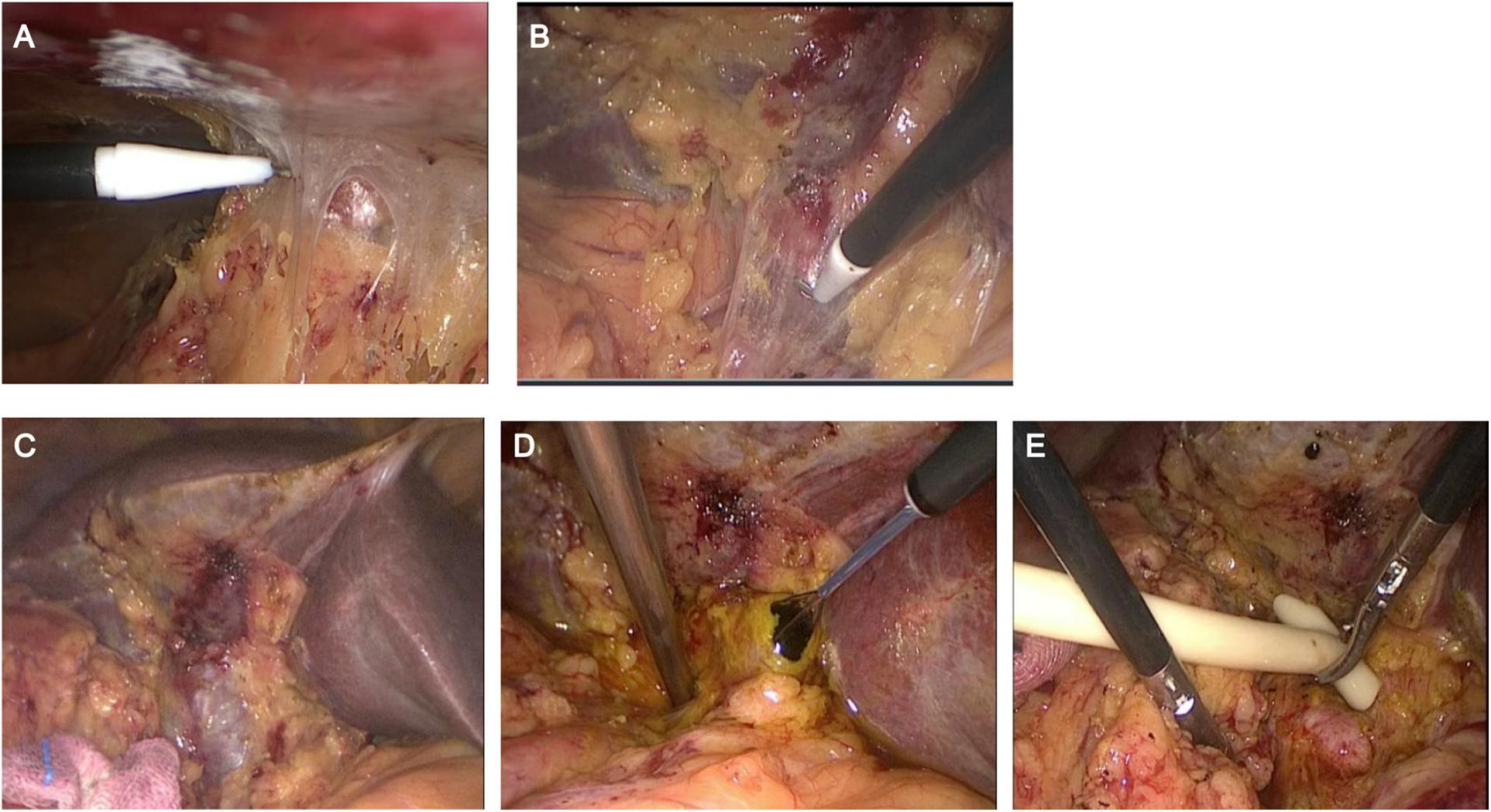
The laparoscopic biliary reoperation procedures. **(A)** Dissection of abdominal adhesions close to the abdominal wall; **(B)** Dissection of hilar adhesions close to the liver; **(C)** Identification of the common bile duct; **(D)** Removal of common bile duct stones under choledochoscopy; **(E)** Placement of T-tube.

The laparoscopic biliary reoperation procedures were displayed as follows: 1) Pneumoperitoneum was established using a Veress needle or under direct vision, ensuring that its position was as far as possible from the original surgical site; 2) After inserting the observation port, the first operative port was established at a site distant from the adhesions if extensive intra-abdominal adhesions were presented. A tissue hook was adopted to dissect the adhesions to create the second operative port. During dissection, the hook was kept as close to the abdominal wall as possible to prevent intestinal injury (Figure 1A); 3) A combination of blunt and sharp dissection was employed, and these instruments were kept as close to the liver to separate the adhesions between the tissues and the liver. The common bile duct was exposed based on its adjacent association with the liver. If the common bile duct was difficult to identify, a bile duct puncture was performed for confirmation. In cases of accidental puncture of the blood vessels, including portal vein, hemostasis was achieved using Prolene suture 5-0 (Figures 1B, C). and 4) A longitudinal incision was made in the anterior wall of the common bile duct. After inserting a choledochoscopy, the stones were removed using a stone basket. For large stones, laser lithotripsy was performed for removal, with lithotripsy time controlled within 2 h. If residual stones remain, they are retrieved postoperatively through the T-tube sinus (Figures 1D, E). All surgeries were performed by the same surgical team.

### Observation indicators

The following indicators were compared between the two groups: operation time, time to first flatus, postoperative pain scores, length of hospital stay, hospitalization costs, and postoperative complications. Additionally, the assessment of the complications was conducted, such as severe nausea and vomiting, bile leakage, intra-abdominal hemorrhage, incision infection, intra-abdominal infection, pulmonary infection, and intestinal/bile duct injury. Serum levels of C-reactive protein (CRP) and prealbumin (PAB) were monitored on postoperative days 1, 3, and 5.

Wound pain was assessed using the Faces Pain Scale – Revised on a scale from 1 to 10 to express the pain intensity. Among them, “0” represented no pain, and “10” indicated the most severe pain, suggesting that the server pain was related to the high score. The first pain assessment was conducted 6 h after anesthesia recovery, followed by assessments every 6 h until 72 h after surgery.

In addition, early postoperative functional recovery indicators were recorded, including time to first ambulation and time to first oral intake (both in hours). Patient satisfaction was evaluated at discharge and at 3 months postoperatively using a four-level scale: very satisfied, satisfied, neutral, and dissatisfied. Quality of life was assessed at the 4-week follow-up using the SF-36 questionnaire, which includes 36 items across 8 dimensions: physical functioning (PF), role physical (RP), bodily pain (BP), general health (GH), vitality (VT), social functioning (SF), role emotional (RE), and mental health (MH). Each domain is scored from 0 to 100, with higher scores indicating better health status. Physical component summary (PCS) and mental component summary (MCS) scores were also calculated. To further evaluate postoperative inflammation, serum interleukin-6 (IL-6) concentrations were measured using enzyme-linked immunosorbent assay (ELISA) on postoperative days 1, 3, 5 and 6 (pg/mL).

### Discharge criteria and follow-up

The discharge criteria were listed as follows: 1) absence of uncomfortable symptoms, including abdominal pain, distension, nausea, or vomiting; 2) ability to eat normally; 3) pain relief at the surgical site or effective pain control with oral analgesics, with gradual improvement; 4) normal passage of gas and stool; 5) bilirubin levels decreased compared to preoperative levels or near normal; 6) well-healed incision without infection; 7) patient consent and desire for discharge.

The follow-up was presented as follows: Patients were followed up weekly for the first 4 weeks after discharge. After 4 weeks, T-tube cholangiography was performed to confirm the absence of residual stones before removing the T-tube (If residual stones were detected, patients were readmitted to remove the stones through the T-tube sinus choledochoscopy at 8 weeks postoperatively). Follow-up was continued every 2 weeks until 12 weeks post-discharge, mainly assessing the patient's eating habit, abdominal symptoms, liver function changes, and passage of gas and stool.

### Statistical analysis

Data were analyzed using SPSS Statistics 25. Postoperative pain scores were measured repeatedly from 0 to 72 h; therefore, a repeated-measures ANOVA was used to assess group effects, time effects, and group × time interactions. When a significant interaction was observed, *post-hoc* independent-samples t-tests with Bonferroni correction were applied to compare the two groups at each time point. Categorical data were compared using the *χ*^2^ test or Fisher's exact test. *P* < 0.05 was considered statistically significant.

## Results

### Basic information

A total of 60 patients were enrolled in this study, including 27 males and 33 females, with ages ranging from 26 to 78 years and an average age of 52.55 ± 14.46 years. Both the ERAS group and the control group consisted of 30 patients each. No significant differences were observed between the two groups in gender, age, time since the first surgery, and the type of initial surgery (*P* > 0.05, Table 2).

**Table 2:** Comparison of basic information between the ERAS and the control groups.

Group	Number of cases	Gender		Age (years)	Time since the first surgery (years)	Type of initial surgery	
		Male	Female			Open surgery	Minimally invasive surgery
ERAS group	30	14	16	53.53 ± 15.19	6.94 ± 4.00	10	20
Control group	30	13	17	51.57 ± 13.88	7.84 ± 4.25	13	17
*χ*^2^/t		0.067		0.523	−0.845	0.635	
*P*		0.795		0.603	0.401	0.425	

Values were presented as mean ± standard deviation or number. ERAS, enhanced recovery after surgery.

## Efficacy analysis

### Postoperative recovery outcomes

The time to first flatus was significantly earlier in the ERAS group than that in the control group. Additionally, the ERAS group exhibited a shorter postoperative length of hospital stay compared to the control group, as well as reduced hospitalization costs (*P* < 0.05, Table 3). Although not statistically significant (*P* = 0.094), the ERAS group had an average operation time approximately 21 min shorter, indicating a trend that should be interpreted cautiously.

**Table 3:** Postoperative recovery outcomes between the ERAS and the control groups.

Group	Number of cases	Time to first flatus (h)	Postoperative length of hospital stay (d)	Operation time (h)	Hospitalization costs (RMB)
ERAS group	30	48.40 ± 6.55	7.53 ± 2.10	2.53 ± 0.61	19,779.68 ± 755.89
Control group	30	72.67 ± 4.61	11.54 ± 2.52	2.73 ± 0.29	22,600.73 ± 974.30
*t*		−16.31	−0.67	−1.702	−12.406
*P*		0.000	0.000	0.094	0.000

ERAS, enhanced recovery after surgery.

### Postoperative pain scores

**Figure 2:**
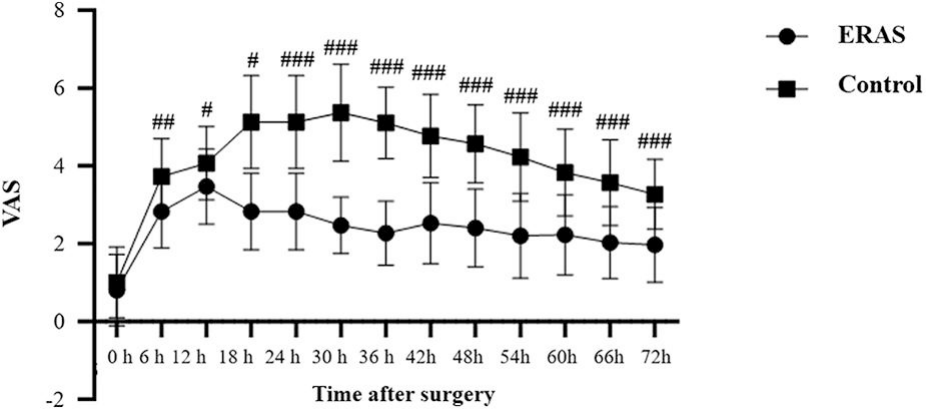
Postoperative pain scores at different time points in the ERAS and control groups. Values are presented as mean ± SD. Between-group differences across time were evaluated using repeated-measures analysis with Bonferroni-adjusted *post-hoc* comparisons. # *P* < 0.05, ## *P* < 0.01 and ### *P* < 0.001 vs. Control group.

Repeated-measures ANOVA revealed significant group and group × time interaction effects, indicating different postoperative pain trajectories between the two groups. *post-hoc* Bonferroni-adjusted t-tests further showed that pain scores at each postoperative time point from 6 h to 72 h were significantly lower in the ERAS group compared with the control group (*P* < 0.05). Pain scores in the ERAS group showed a continuous downward trend throughout the observation period, whereas the control group maintained relatively higher levels (Figure 2).

### Comparison of postoperative serum indicators

**Figure 3:**
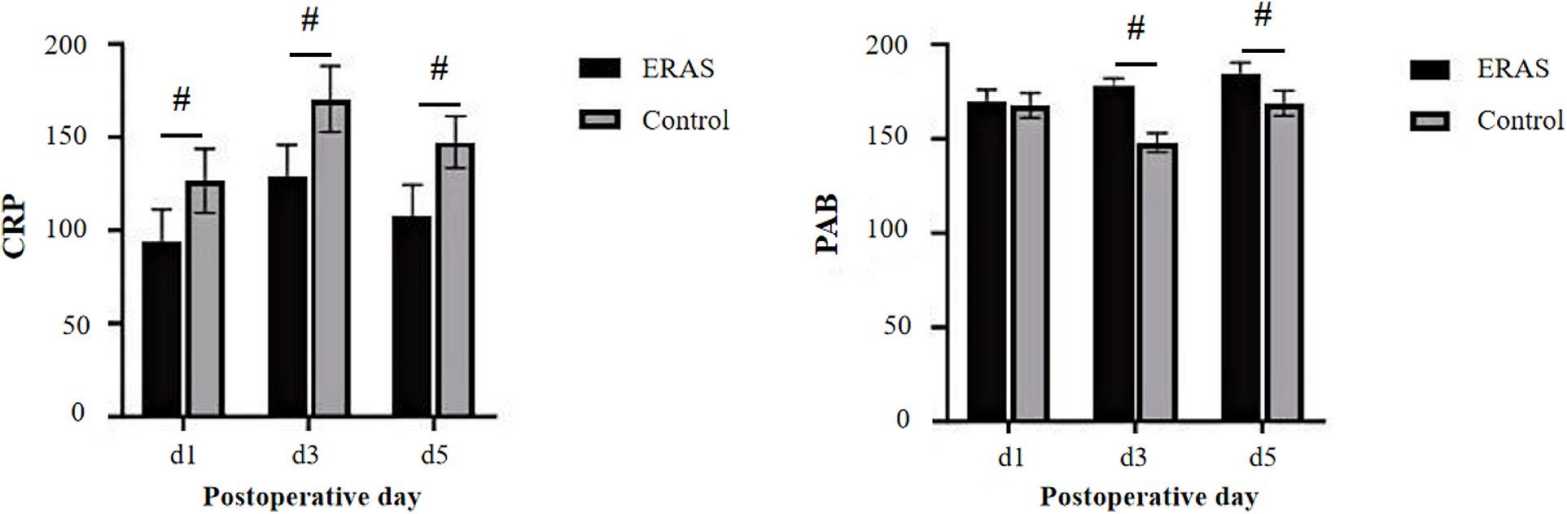
Values are presented as mean ± SD. Comparison of postoperative serum indicators between the ERAS and the control groups. CRP, C-reactive protein; PAB, prealbumin; ERAS, enhanced recovery after surgery. # *P* < 0.05 vs. Control group.

In terms of postoperative inflammatory stress, both serum IL-6 and CRP levels increased significantly on postoperative day 1 and peaked on day 3 in both groups, then markedly declined by day 5 and day 6. At all time points after surgery, IL-6 and CRP levels in the ERAS group were significantly lower than those in the control group (*P* < 0.05, Figure 3), indicating a milder inflammatory response and more rapid resolution of systemic inflammation. Regarding nutritional status, no significant difference in PAB levels was observed between the two groups on postoperative day 1. However, PAB levels were significantly higher in the ERAS group than in the control group on days 3 and 5 postoperatively (*P* < 0.05), suggesting that the ERAS protocol contributed to enhanced early postoperative nutritional recovery.

### Comparison of postoperative indicators and satisfaction

As shown in Table 4, the ERAS group achieved significantly faster postoperative recovery, with earlier time to first ambulation (27.67 ± 5.46 h vs. 39.67 ± 6.78 h, *P* < 0.001) and first oral intake (16.10 ± 4.29 h vs. 24.30 ± 5.97 h, *P* < 0.001) compared to the control group. Patient satisfaction was also higher in the ERAS group at discharge (*χ*^2^ = 7.938, *P* = 0.019) and at 3 months postoperatively (*χ*^2^ = 17.452, *P* < 0.001), with more patients reporting very satisfied or satisfied responses at both time points.

**Table 4:** Comparison of postoperative indicators and satisfaction between the two groups.

Variable	ERAS	Control	t/*χ*2	*P*
First time out of bed	27.67 ± 5.46	39.67 ± 6.78	−7.547	<0.001
First time eating	16.10 ± 4.29	24.30 ± 5.97	−6.11	<0.001
Day of discharge			7.938	0.019
Very satisfied	8 (26.7)	5 (16.7)		
Satisfied	18 (60.0)	11 (36.7)		
Normal	4 (13.3)	14 (46.7)		
Unsatisfied	0 (0.0)	0 (0.0)		
3 months after surgery			17.452	<0.001
Very satisfied	19 (63.3)	5 (16.7)		
Satisfied	11 (36.7)	17 (56.7)		
Normal	0 (0.0)	8 (26.7)		
Unsatisfied	0 (0.0)	0 (0.0)		

### Comparison of SF-36 scores

At 4 weeks postoperatively (Table 5), patients in the ERAS group showed significantly higher scores than those in the control group across multiple SF-6 domains, including physical functioning (PF: 85.67 ± 2.52 vs. 78.10 ± 4.38, *P* < 0.001), role physical (RP: 83.40 ± 3.58 vs. 75.37 ± 4.41, *P* < 0.001), bodily pain (BP: 82.73 ± 4.27 vs. 72.20 ± 3.09, *P* < 0.001), general health (GH: 85.00 ± 5.00 vs. 77.73 ± 4.11, *P* < 0.001), and vitality (VT: 85.03 ± 3.85 vs. 76.13 ± 4.42, *P* < 0.001). No significant differences were observed in SF, RE, or MH between groups (*P* > 0.05). The PCS score was significantly higher in the ERAS group (51.36 ± 1.68 vs. 46.00 ± 2.08, *P* < 0.001), while no difference was noted in the MCS score (*P* = 0.914).

**Table 5:** Comparison of SF-36 scores between the two groups 4 weeks after surgery.

SF-36 domain	ERAS	Control	*t*	*P*
PF	85.67 ± 2.52	78.10 ± 4.38	8.197	0.000
RP	83.40 ± 3.58	75.37 ± 4.41	7.753	0.000
BP	82.73 ± 4.27	72.20 ± 3.09	10.953	0.000
GH	85.00 ± 5.00	77.73 ± 4.11	6.147	0.000
VT	85.03 ± 3.85	76.13 ± 4.42	8.308	0.000
SF	79.67 ± 7.24	79.03 ± 4.00	0.419	0.677
RE	80.37 ± 6.92	78.90 ± 4.55	0.970	0.336
MH	80.30 ± 7.28	78.87 ± 4.18	0.935	0.355
PCS	51.36 ± 1.68	46.00 ± 2.08	10.969	0.000
MCS	51.41 ± 3.49	51.49 ± 2.35	−0.109	0.914

### Postoperative complications

For postoperative complications, there were no intestinal/bile duct injuries, intra-abdominal infection, or postoperative intra-abdominal hemorrhage in the ERAS and the control groups. Additionally, no significant differences were observed between the two groups in bile leakage, incision infection, or pulmonary infection (*P* > 0.05). However, the response to severe nausea and vomiting was markedly less in the ERAS group compared to the control group (*P* < 0.05, Table 6).

**Table 6:** Postoperative complications between the ERAS and control groups.

Complication	ERAS	Control	χ^2^	*P*
Bile leakage	1 (3.3)	3 (10.0)	1.071	0.301
Lung infection	2 (6.7)	3 (10.0)	0.218	0.64
Wound infection	1 (3.3)	4 (13.3)	1.964	0.161
Severe nausea and vomiting	3 (10.0)	11 (36.7)	5.963	0.015
Total complications	6 (20.0)	21 (70.0)	15.152	<0.001

ERAS, enhanced recovery after surgery.

All patients were cured and discharged without perioperative mortality or loss to follow-up. During the follow-up period, none of the patients required reoperation or rehospitalization due to postoperative complications.

## Discussion

ERAS refers to a set of perioperative management measures that are rigorously supported by evidence-based medicine to accelerate postoperative rehabilitation. In this study, the ERAS concept was adopted for the patients with extrahepatic bile duct stones undergoing reoperation in perioperative management. The results demonstrated that clinical rehabilitation indicators in the ERAS group were superior to those of patients treated with traditional perioperative management.

ERAS encompasses preoperative, intraoperative, and postoperative phases, and the adverse effects caused by various stressors should be avoided in each phase (8). In this study, the ERAS group received relevant education from the patient's hospitalization. Detailed information about the patient's condition and expected outcomes was provided by medical staff, ensuring that patients and their family members were well-informed about the entire treatment process. Additionally, appropriate guidance by nursing staff was offered to help patients quickly acclimate to their surroundings, thereby effectively alleviating anxiety and tension, increasing the subjective initiative of patients, and promoting their rehabilitation.

For preoperative preparation, the traditional concept holds that surgery for recurrent extrahepatic bile duct stones is an open surgery, and routine preoperative intestinal preparation should be performed for accidental intestinal injuries. However, this preparation, on the one hand, can increase patient anxiety and discomfort. On the other hand, it may lead to multiple conditions such as electrolyte imbalance and ectopia of intestinal flora, thereby increasing the incidence of postoperative complications and delaying the postoperative rehabilitation of patients (9, 10). Furthermore, preoperative placement of a nasogastric tube for decompression can not only cause throat discomfort but also hinder the normal physiological recovery of gastrointestinal function postoperatively (11, 12). In this study, the incidence of adverse events, such as abdominal distension, was not increased in the ERAS group without routine cleaning enema preoperatively. Additionally, no adverse events, including aspiration, occurred without nasogastric tube placement in the ERAS group. Furthermore, for gastric emptying to prevent aspiration, the ERAS group received oral administration of 10% glucose solution 2 h preoperatively, which was different from the traditional perioperative management requiring 12 h of fasting and 6 h of water deprivation. Such a method not only alleviated thirst, anxiety, hunger, and hypoglycemia but also increased liver glycogen reserves and reduced the incidence of insulin resistance (13, 14). In this study, no serious complications, such as aspiration, were caused by this method in the ERAS group. In addition to traditional recovery parameters, patients in the ERAS group achieved significantly earlier ambulation and oral intake, reflecting faster early postoperative functional recovery. Moreover, patient satisfaction at both discharge and 3-month follow-up was notably higher in the ERAS group. These findings indicate that ERAS not only accelerates physiological recovery but also improves the patient's subjective experience and acceptance of treatment.

Control of intraoperative fluid volume and rate, alongside maintaining body temperature, are critical factors for postoperative recovery (15). Intraoperative hypothermia exacerbates the body's stress responses, and the process of rewarming from hypothermia may lead to coagulation abnormalities and increase the risk of postoperative infections. Furthermore, such a process can further increase the cardiovascular burden to result in perioperative cardiovascular events. Additionally, the infusion of large volumes of hypothermal liquid intraoperatively can not only increase cardiopulmonary loads and interstitia fluid retention, but also decrease the liver metabolism metabolic function and coagulation function. Strict intraoperative fluid control and prevention of hypothermia can prevent the onset of aforementioned conditions, reduce the above risks, and promote the recovery of gastrointestinal function.

Regarding postoperative recovery, considering that reoperation is time-consuming, urinary catheters were placed in both groups. However, patients in the ERAS group did not require bladder function training for early removal of the catheter, and no cases of reinsertion were observed. In clinical practice, the main role of drainage tube placement was to drain intra-abdominal fluid accumulation. An abdominal drainage tube was routinely placed to observe whether there is intra-abdominal bleeding or bile leakage, particularly in patients undergoing primary suture of the common bile duct. Nevertheless, the retention of a drainage tube could increase the likelihood of postoperative pain and infection around the drainage site to some extent and exacerbate psychological and physiological burden. Such results could further affect the patient's motivation for early ambulation and oral intake, ultimately prolonging recovery time. Therefore, the postoperative drainage tube should be removed as early as possible in the absence of active bleeding, bile leakage, or excessive exudate. This study revealed that early removal of the abdominal drainage tube did not result in an increased incidence of postoperative intra-abdominal infections or bleeding; however, these treatments were based on precise surgical suturing techniques and minimal mild bile duct inflammation and edema, and the serious consequences caused by blind removal of the drainage tube should be avoided. Recent studies have emphasized the value of patient-centered outcomes, such as quality of life and satisfaction, in evaluating surgical strategies. In this study, ERAS patients scored significantly higher in several SF-36 domains—including physical functioning, vitality, and general health—at 4 weeks postoperatively, consistent with earlier reports that ERAS can improve early postoperative quality of life in open surgery.

Early ambulation postoperatively is beneficial for the recovery of gastrointestinal and pulmonary function and reduces the incidence of venous thrombosis. Effective pain management is a prerequisite for early mobilization and oral intake. A multimodal analgesia approach, including scheduled non-opioid agents combined with patient-controlled intravenous opioids, was employed in the ERAS group, whereas the control group received standard stepwise analgesia with on-demand non-opioid medications and rescue opioids. However, our ERAS protocol did not include advanced abdominal wall blocks and instead relied on wound infiltration plus systemic multimodal analgesia, which may have limited the full analgesic potential of contemporary ERAS pathways. This study demonstrated that postoperative wound pain was significantly better in the ERAS group compared to the control group (*P* < 0.05). Additionally, early ambulation can, to some extent, promote gastrointestinal peristalsis and prevent complications caused by long-term bedridden, such as hypostatic pneumonia, and lower extremity venous thrombosis (16). Furthermore, early postoperative oral intake, on the one hand, can accelerate the recovery of gastrointestinal peristalsis and maintain the integrity of the intestinal mucosal barrier; on the other hand, it can reduce the volume and duration of infusion, improve hepatic synthetic function and enhance overall nutritional status (17). PAB, synthesized by hepatocytes, has a short half-life of approximately 1.9 days. Hence, the measurement of PAB level in plasma is highly sensitive for understanding protein malnutrition and liver dysfunction. In this study, the PAB levels were comparable between the two groups on postoperative day 1. However, the control group exhibited a significant decrease in the PAB levels on postoperative days 3 and 5 compared to the ERAS group, caused by the continuous consumption of surgical stress and trauma and the inability to resume early oral intake. Therefore, in this study, postoperative time to first flatus and nutritional status was markedly better in the ERAS group than those in the control group, greatly greatly promoting recovery and improving nutritional status. In our protocol, IL-6, CRP, PAB and postoperative pain scores were intentionally measured only at postoperative time points as dynamic indicators of surgical stress and recovery, rather than as baseline characteristics. Although all patients underwent routine preoperative laboratory assessments and no obvious imbalance between the two groups was identified, the lack of detailed preoperative IL-6/CRP/PAB values and standardized pain scores means that a certain degree of residual confounding by preoperative status cannot be completely excluded. Although operation time did not reach statistical significance, the ERAS group had an average reduction of approximately 21 min. This trend may reflect differences in operative complexity and is acknowledged as a potential confounder that could influence postoperative recovery.

Regarding complications, this study revealed that there were no significant differences between the ERAS and control groups in the incidence of postoperative incision infection, pulmonary infection, or bile leakage. However, the incidence of severe postoperative nausea and vomiting was remarkably lower in the ERAS group. This reduction in nausea and vomiting may be attributed to the ERAS protocol's multimodal analgesic strategy, which relies primarily on non-opioid medications and consequently lowers overall opioid exposure, a recognized risk factor for postoperative nausea and vomiting. Additionally, postoperative serum CRP levels, an indicator of postoperative serological indicators, were remarkable lower in the ERAS group relative to the control group. This indicator, to some extent, reflects the body's inflammation and stress response, suggesting that the ERAS protocol in this study effectively mitigated postoperative inflammation and adverse stress response, thereby benefiting patient recovery.

## Conclusion

This study explored the application of the ERAS concept in laparoscopic biliary reoperation for extrahepatic bile duct stones and achieved some positive results. However, there are some limitations in this study. For instance, only 60 patients were included in this study, which is a small sample size and may affect the generalizability of the findings. Large-scale multicenter studies are needed to further validate the effectiveness of the ERAS concept. The application of the ERAS concept in the surgery for extrahepatic bile duct stones should be further validated and refined.

## Data Availability

The original contributions presented in the study are included in the article/Supplementary Material, further inquiries can be directed to the corresponding authors.
